# Analysis of Polyphenolic Composition of a Herbal Medicinal Product—Peppermint Tincture

**DOI:** 10.3390/molecules25010069

**Published:** 2019-12-24

**Authors:** Agnieszka Bodalska, Adam Kowalczyk, Maciej Włodarczyk, Izabela Fecka

**Affiliations:** Wroclaw Medical University, Faculty of Pharmacy, Department of Pharmacognosy and Herbal Medicines, ul. Borowska 211, 50-556 Wroclaw, Poland; agnieszka.bodalska@student.umed.wroc.pl (A.B.); adam.kowalczyk@umed.wroc.pl (A.K.); maciej.wlodarczyk@umed.wroc.pl (M.W.)

**Keywords:** *Mentha* × *piperita*, polyphenols, flavonoids, caffeetannins, phenolic acids, herbal medicinal product, peppermint tincture

## Abstract

The pharmacological activity of peppermint leaf (*Menthae piperitae folium*) for medical use is mainly attributed to the presence of essential oil, which, according to the European Pharmacopoeia (Ph. Eur.), should constitute not less than 12 mL/kg of raw material. The content of polyphenols in peppermint-based preparations, except peppermint leaf dry extract, has not yet been considered as an essential parameter in the pharmacopeial assessment of peppermint quality. This study concerns the evaluation of the presence of representatives of polyphenolic compounds in 23 commercial peppermint tinctures (ethanolic extracts) purchased in pharmacies in Poland. The non-volatile polyphenolic fraction was investigated, and the presence of flavonoids and phenolic acids was quantified. High performance liquid chromatography coupled with a diode-array detector (HPLC-DAD) and an electrospray ionization mass spectrometer (U(H)PLC-ESI-MS) were used in the experiment. The study showed that eriocitrin, luteolin-7-*O*-rutinoside, and rosmarinic acid were the main polyphenolic components of the peppermint tinctures, as previously reported for peppermint leaf. Despite this, the research shows the extremely diverse content of the mentioned compounds in analyzed commercial medicinal products. In light of these results, it seems that the pharmacopeial assessment for the peppermint leaf (Ph. Eur.) and peppermint tincture (Polish Pharmacopoeia (FP)) requires correction and supplementation.

## 1. Introduction

The importance of polyphenols (flavonoids, phenolic acids, depsides, stilbenes, lignans, tannins, etc.), common compounds in the plant kingdom, is widely known in health care. These bioactive secondary metabolites have been an inexhaustible source of scientific research including the determination of their chemical structure and diverse biological properties [1]. The most commonly used polyphenols in treatment are flavonoids (2-phenyl-benzo-gamma-pyrone) as well as phenolic acids and their esters (depsides) [2]. *Mentha* × *piperita* L. fam. Lamiaceae (syn. Labiatae), a hybrid which originated from crossing the two species, *Mentha aquatica* L. and *Mentha spicata* L., has been used in medicine since the 13th century in the form of infusions, tinctures, dry and liquid extracts, and essential oil or its constituents (menthol, menthone, and other monoterpenes) mostly as a cholagogue, choleretic, digestive, carminative, and spasmolytic in gastrointestinal disturbances and against respiratory system diseases [1,3]. This plant is cultivated in Europe (mainly England and Germany), North America, and other countries in the temperate climate zone [4]. It is classified as a traditional herbal medicine with indications based on long-standing use for relief of digestive disorders, gastritis, enteritis biliary disorders, diarrhea, skin disorders and minor wounds, pain and inflammation, as well as coughs and colds [5]. Both peppermint leaf (*Menthae piperitae folium*) and peppermint oil (*Menthae piperitae aetheroleum*) are official medicines in Europe (European Pharmacopoeia, Ph. Eur.; EMA/HMPC—European Medicines Agency/Committee on Herbal Medicinal Products) [6,7,8] and are listed in the U.S. National Formulary [9]. Peppermint oil, spirit, and water have official monographs in the United States Pharmacopeia (USP) [9]. The monograph of peppermint tincture (*Menthae piperitae tinctura cum Menthae piperitae aetheroleo*) is listed in the Polish Pharmacopoeia (FP) [10], and obtained by one day maceration of 50 parts of peppermint leaf (pulverized and sieved through sieve no. 45) with 927 parts of 96% ethanol and 73 parts of water. After filtration, 50 parts of *Menthae piperitae aetheroleum* is added. *Menthae piperitae tinctura cum Menthae piperitae aetheroleo* is usually used orally in dyspepsia, intestinal colic, and flatulence.

Among peppermint leaf polyphenols, eriocitrin with a concentration range of 6%–15%, is the dominant flavonoid glycoside, accompanied by luteolin-7-*O*-rutinoside, luteolin-7-*O*-glucuronide, luteolin-7-*O*-glucoside, hesperidin, and diosmin [4,11,12]. The second group of polyphenols, equally important and crucial for health, comprises phenolic acids (especially caffeic acid) and their esters (depsides), which, as studies have shown, also occur in other species from the Lamiaceae family [13]. For this reason, the main representative of phenolic acids in this family—rosmarinic acid—has been called labiataetannin or caffeetannin [14,15]. Amoah and co-workers [16] conducted the first comprehensive review of many articles published in the years 1990–2015 regarding the current knowledge about this compound, including in vitro, in vivo, and clinical trials. Among the numerous indications for the use of rosmarinic acid in therapy, the most important are studies on its exceptional antiphlogistic, antioxidant, and free-radical scavenging properties (higher than other hydroxycinnamic acid derivatives), antimicrobial and antiviral action, as well as those suggesting the possible beneficial role of this compound in the treatment of depression. Geuenich and co-workers [17] describe rapid and potent action to reduce human immunodeficiency virus (HIV)-1 infectivity by aqueous extracts of three well-known lamiaceous species, including *Mentha piperita* L. Based on this study the authors suggested that those extracts may be the starting point for possible development of new virucidal microbicides.

Polyphenols are considered as the main free-radical scavenging compounds in *Menthae piperitae folium* preparations; thus, many assessments on antioxidant properties of peppermint extracts have been conducted [18,19,20,21,22]. Due to the dynamic discovery of new biological properties of polyphenols important for medicine, a resumption of interest in peppermint leaf in medicine has been observed. Pharmacological studies, especially from the last 20 years, have revealed a number of newer biological effects of peppermint leaf including antiallergic, nociceptive, anti-inflammatory, and iron (II) chelating properties (which is an important factor of the antioxidant process), as well as beneficial effects in diseases of the vascular and nervous system (e.g., mental disorders) [23,24]. Animal studies suggest the possibility of using peppermint preparations to treat depression, a disease which according to the World Health Organization (WHO) forecast may become in the 2030s one of the most common diseases all over the world [25,26]. Researchers have proven that *Menthae piperitae folium* extract has an impact on the monoaminergic system, thus causing antidepressant-like action. Although many researchers consider essential oil as an anti-depressant factor, Maleki and co-workers [27] claim that the polyphenolic fraction and its main constituents—rosmarinic acid, eriocitrin, and luteolin-7-*O*-rutinoside—may also be responsible for this biological action. In addition, in non-volatile toxicity studies, the absolute safety of peppermint use ad libitum has been demonstrated. There were no side effects or any cases of mortality among experimental animals, even at doses 10 times higher than the initial dose (from 400 to 4000 mg per kg of body weight) [28]. When considering peppermint safety, most data concern volatile oil, menthol, menthone, and other possible constituents such as pulegone and menthofuran. Despite this, according to the overall toxicity assessment, peppermint and its preparations are considered as safe medicines [29].

The aim of this study is to evaluate the concentration of polyphenolic compounds (flavonoids and phenolic acids—caffeetannins) in different commercial peppermint tinctures and to present the most important components from a pharmacological point of view. The variability of polyphenol concentration levels in these preparations has not been studied before.

## 2. Results

**Figure 1:**
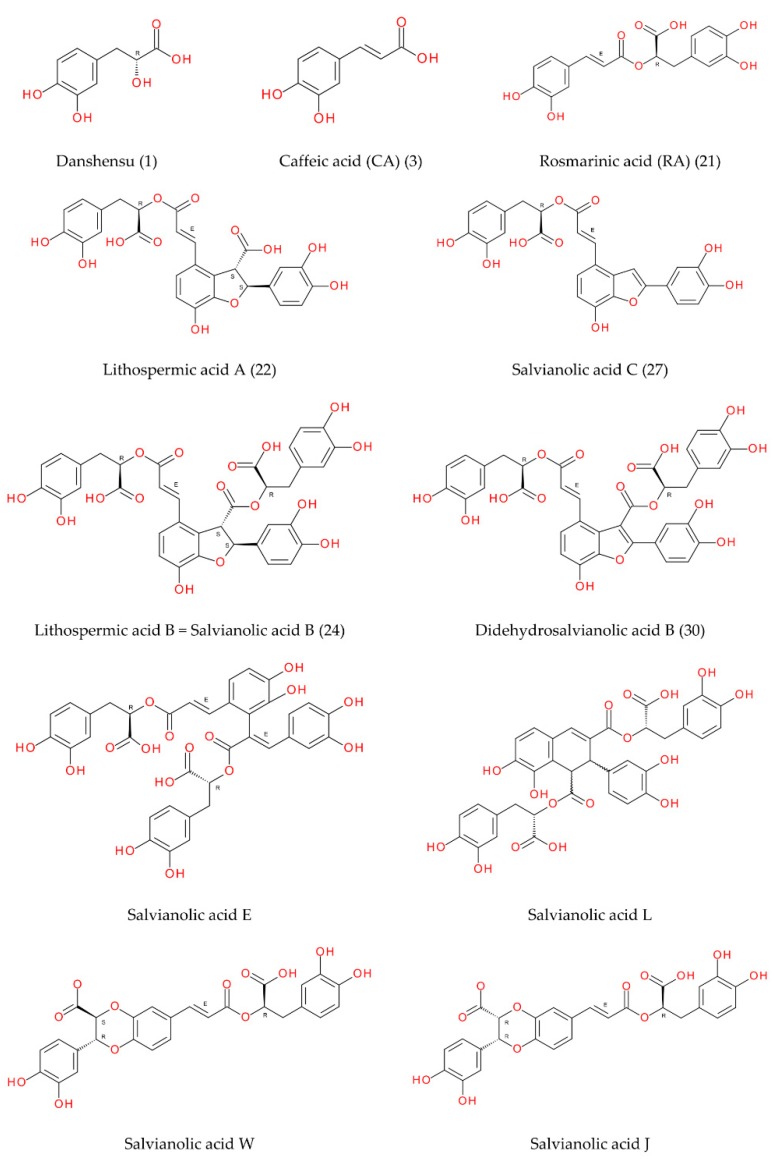
Caffeic acid derivatives.

**Figure 2:**
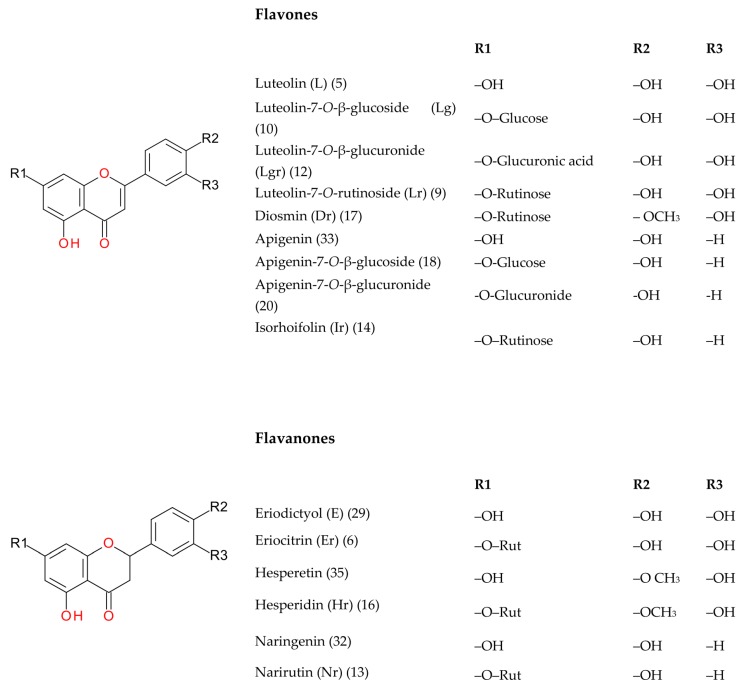
Flavonoids.

**Figure 3:**
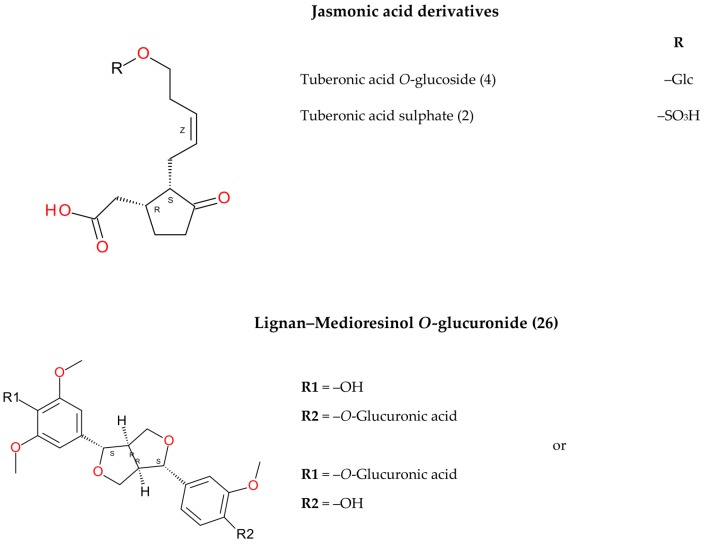
Other compounds.

The investigation into peppermint tinctures’ (23 commercial medicinal products) polyphenolic composition shows the presence of 35 compounds (among them 12 phenolic acids—caffeetannins, 12 flavones, eight flavanones, two jasmonic acid derivatives, and one lignan), of which 10 were determined quantitatively. Chemical structures of identified peppermint components are presented in Figure 1, Figure 2 and Figure 3.

The electrospray ionization mass spectrometer coupled with ultra high performance liquid chromatography (U(H)PLC-ESI-MS) results together with MS/MS fragmentations are presented in Table 1. This analysis has not only confirmed the presence of well-known polyphenols, but also revealed the occurrence of components in peppermint leaf preparations not previously described. 

**Figure 4:**
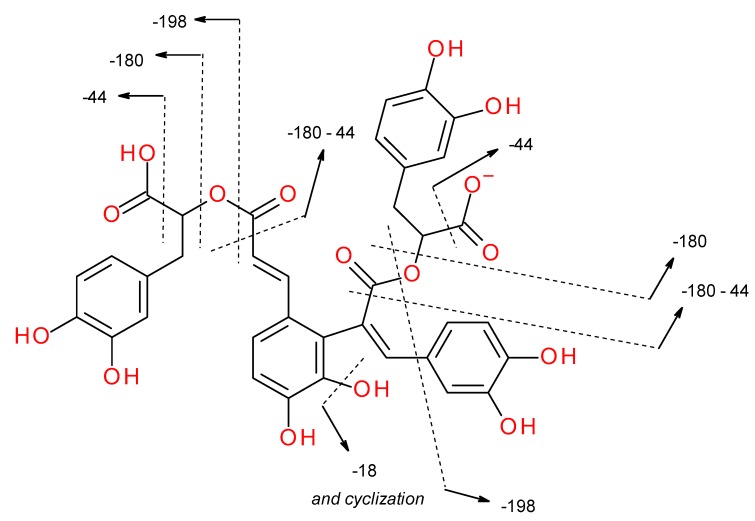
Proposed MS/MS fragmentation pathway of caffeetannins (e.g., salvianolic acid E (tetramer)).

In addition to caffeic acid (peak 3) and rosmarinic acid (peak 21) this research has shown the presence of ten other caffeic acid esters (peaks 1, 7, 15, 19, 22, 24, 25, 27, 30, 34) known as lithospermic acids or salvianolic acids. Caffeic acid, rosmarinic acid, lithospermic acid A (peak 22), and lithospermic acid B (peak 24) were identified using authentic standards or herbal reference materials (Salvia miltiorrhiza Bunge, Melissa officinalis L.) and literature data [32,34,35]. Interpretation of data from MS/MS experiments for remaining peppermint caffeetannins is presented in Table 2. Peak 1 with pseudo-molecular ion at *m/z* 197.0454 (fragment ions at 181 and 137) was identified as salvianic acid A (syn. danshensu). Peak 7 with [M − H]^−^ at *m/z* 537.1048, isobaric to peak 22 was identified as salvianolic acid J or W (lithospermic acid A isomers) [31]. Three isobaric peaks, 15 with *m/z* 717.1442 (fragment ions at 475 [M-242 − H]^−^, 431 [M-286 − H]^−^, 339 [M-378 − H]^−^, 321 [M-396 − H]^−^, and 295 [M-422 − H]^−^), 19 with *m/z* 717.1440 (fragment ions at 339, 321, and 295), and 25 with *m/z* 717.1392 were specified as isomers of lithospermic acid B (syn. salvianolic acid B) salvianolic acids E, L or others [30,32,41]. Peak 27 with a pseudo-molecular ion at *m/z* 491.0988 produced the fragment of 311 [M-180 − H]^−^ and 293 [M-198 − H]^−^ which was characterized as salvianolic acid C [35]. On the basis of pseudo-molecular (*m/z* 715.1293) and fragment ions (*m/z* 337 [M-378 − H]^−^, 319 [M-396 − H]^−^ and 293 [M-422 − H]^−^), peak 30 was tentatively identified as didehydrosalvianolic acid B [36]. Peak 34 (*m/z* 387.1092) was specified as ethyl rosmarinate (probably a second artefact created by esterification with ethanol) [37]. Fragment ions of detected caffeetannins were formed after the neutral loss of the following molecules: water (−18 Da), carbon dioxide (−44 Da), dehydroxysalvianic acid A residue (syn. dehydroxydanshensu residue; C_9_H_8_O_4_, −180 Da), and salvianic acid A (syn. danshensu; C_9_H_10_O_5_, −198 Da) (Table 2) [34]. Proposed MS/MS fragmentation pathway of caffeic acid oligomers (e.g., salvianolic acid E) is showed in Figure 4. 

Twelve compounds, identified as flavones (peaks 5, 9, 10, 12, 14, 17, 18, 20, 23, 28, 31, 33) were eluted between 10.44 and 16.10 min. Among them, luteolin-7-*O*-rutinoside, luteolin-7-*O*-β-glucoside, luteolin-7-*O*-β-glucuronide, isorhoifolin, diosmin, apigenin-7-*O*-β-glucoside, luteolin, and apigenin were identified on the basis of the comparison of chromatographic, UV-Vis, and MS data with corresponding data for authentic standards. Peak 5 with a pseudo-molecular ion at *m/z* 637.1053 and fragment ion at 285 [M-176-176 − H]^−^ was initially elucidated as luteolin-*O*-diglucuronide [38]. Peak 20 (*m/z* 445.0762) was detected as apigenin-7-*O*-glucuronide [42]. Additional minor components of peppermint tincture with a pseudo-molecular ion at *m/z* 461.0723 (peak 23) and 489.1037 (peak 28) were detected as tetrahydroxyflavone derivatives, presumably scutellarin-*O*-glucuronide and an ethyl ester of luteolin-7-*O*-β-glucuronide. Peak 28 was probably an artefact created by esterification of luteolin-7-*O*-β-glucuronide with ethanol.

Flavanones (peaks 6, 8, 11, 13, 16, 29, 32, 35) were detected on the U(H)PLC chromatograms between 11.13 and 16.35 min. Six peaks (6, 13, 16, 29, 32, 35) were identified as eriocitrin, narirutin, hesperidin, eriodictyol, naringenin, and hesperetin, respectively. Their fragmentation, t_R_ and UV-Vis spectra corresponded to proper parameters of authentic standards and literature data [38]. Components with pseudo-molecular ions at 449.1088 (peak 8) and 463.0882 (peak 11) and fragment ion at 287 *m/z* were tentatively specified as eriodictyol derivatives, eriodictyol-7-*O*-glucoside and eriodictyol-7-*O*-glucuronide [38,39].

Peaks 2 and 4 with pseudo-molecular ions at *m/z* 305.0700 and 387.1661 were tentatively characterized as 12-hydroxyjasmonic acid derivatives, tuberonic acid sulphate and tuberonic acid *O*-glucoside, respectively [40]. Peak 26 (*m/z* 563.2124, fragment ion: 387) was described as a medioresinol derivative [38,40].

**Figure 5:**
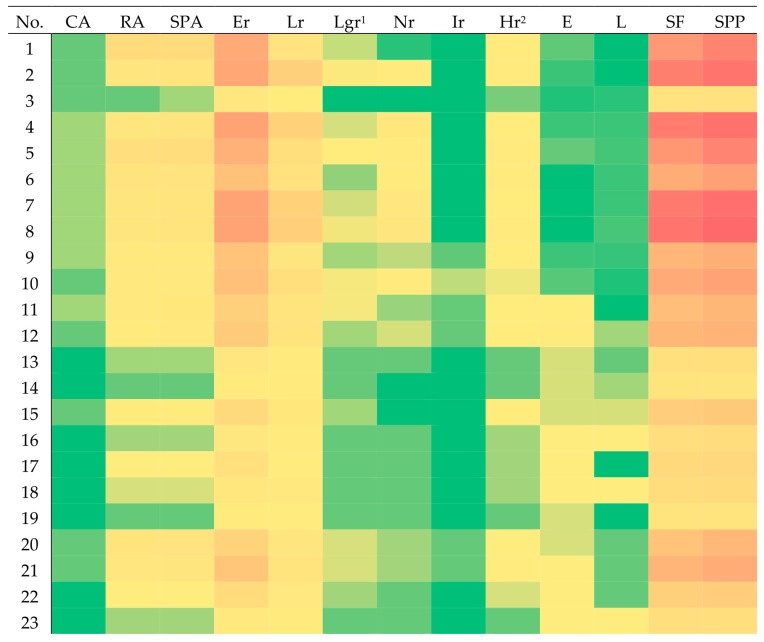
Heatmap of content of determined polyphenolic compounds in peppermint tinctures. CA, caffeic acid; RA, rosmarinic acid; Er, eriocitrin; Lr, luteolin rutinoside; Lgr, luteolin glucuronide (luteolin glucuronide + luteolin glucoside calculated as Lgr); Nr, narirutin; Ir, isorhoifolin; Hr, hesperidin (hesperdin + diosmin calculated as Hr); E, eriodictyol; L, luteolin; SPA, sum of polyphenolic acids; SF, sum of flavonoids; SPP, sum of polyphenols; higher concentration: red, lower concentration: green.

**Figure 6:**
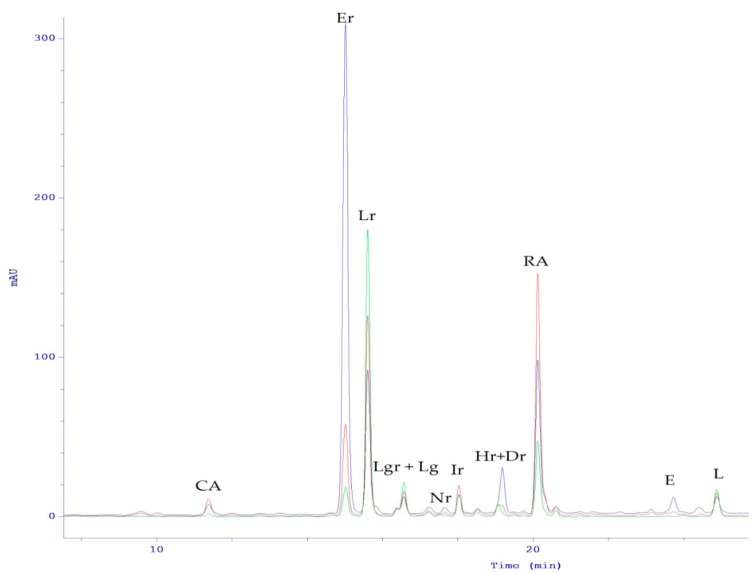
Typical HPLC chromatograms of peppermint tincture at 280 (blue line), 320 (red line), and 360 nm (green line). CA, caffeic acid; Er, eriocitrin; Lr, luteolin rutinoside; Lgr, luteolin glucuronide; Lg, luteolin glucoside; Nr, narirutin; Ir, isorhoifolin; Hr, hesperidin; Dr, diosmin; RA, rosmarinic acid; E, eriodictyol; L, luteolin.

Quantification of principal polyphenols was carried out by the high performance liquid chromatography coupled with a diode-array detector (HPLC-DAD) method described previously by Fecka and Turek [43]. The HPLC-DAD method was re-validated and corresponding data for ten authentic standards are presented in Table 3. Figure 5 shows the heatmap of polyphenol content in peppermint tinctures. Detailed contents of caffeic acid (CA), rosmarinic acid (RA), eriocitrin (Er), luteolin rutinoside (Lr(+Lg), luteolin glucuronide + luteolin glucoside calculated as Lgr), luteolin glucuronide (Lgr), narirutin (Nr), isorhoifolin (Ir), hesperidin (Hr(+Dr), hesperdin + diosmin calculated as Hr), eriodictyol (E), and luteolin (L) and sums of quantified phenolic acids (SPA, CA + RA), flavonoids (SF, Er + Lr + Lgr + Nr + Ir + Hr + E + L), and all polyphenols (SPP) are compared in Table A1. Compound contents are expressed as mg per 1 mL of peppermint tincture. Lg and Lgr as well as Dr and Hr were eluted together, thus the amounts of these components were quantified together. Lithospermic acids, salvianolic acids, and other minor caffeic acid esters were not included in the calculations as their concentrations were below the quantification limit (QL) for rosmarinic acid. Chlorogenic acid was not present at all. Typical HPLC chromatograms of peppermint tincture at 280, 320, and 360 nm are presented in Figure 6.

The predominant phenolic acid derivative was rosmarinic acid, with an average concentration of 0.14 ± 0.12 mg/mL. The highest amount, 0.44 mg/mL, was detected in sample no. 1. Average caffeic acid concentration was 0.01 mg/mL, while the highest observed value was 0.02 mg/mL.

Among analyzed flavanones, eriocitrin (eriodictyol-7-*O*-rutinoside) showed the highest concentration, up to 1.77 mg/mL and an average of 0.81 ± 0.60 mg/mL. The highest amount of Er was recorded in sample no. 7. Other flavanone glycosides, like hesperidin (calculated as the sum with diosmin, because those compounds were eluted together) and naringenin-7-*O*-rutinoside, had respectively 0.04 and 0.05 mg/mL on average. The mean concentration of eriodictyol, eriocitrin aglycone, was 0.03 ± 0.02 mg/mL. The study showed that the most common flavone was luteolin-7-*O*-rutinoside with a mean concentration of −0.27 ± 0.23 mg/mL on average. The highest Lr level was assessed in product no. 8. The average concentration of luteolin-7-*O*-β-glucuronide was 0.02 ± 0.01 mg/mL. Due to a similar retention time, the content of luteolin-7-*O*-β-glucoside was evaluated together with Lgr. The average content of aglycone luteolin was 0.01 ± 0.01 mg/mL. The concentration of isorhoifolin (apigenin-7-*O*-rutinoside) and other flavones and flavanones was in most cases below QL, but its presence was confirmed by U(H)PLC-ESI-MS analysis. The mean sum of phenolic acids (SPA) in analyzed peppermint tinctures was calculated as 0.15 ± 0.12 mg/mL. The average flavonoid sum (SF) and average sum of all quantified polyphenols (SPP) was 1.24 ± 0.87 and 1.39 ± 0.97 mg/mL, respectively. Product no. 8 had the highest values of SF and SPP, while the lowest values were observed in product no. 14.

## 3. Discussion

*Mentha* × *piperita* L. is among the most popular plants of temperate climate countries, where it is cultivated for medicinal purposes. Peppermint leaf (*Menthae piperitae folium*) is obtained from crops and used for various types of diseases. As an official medicine peppermint was mentioned for the first time in the 13th century in the Icelandic Pharmacopeia [24]. Essential oil, whose content in the raw material should not be less than 12 mL/kg, is considered a substance that determines the versatile activity of this plant. In the literature over the last 25 years, attention has been drawn to the non-volatile components of peppermint leaf, mainly polyphenols, primarily flavonoids and phenolic acids—caffeetannins, as pharmacologically active substances. Studies have proved that out of several peppermint extracts prepared with different solvents, aqueous and alcoholic extracts possess the highest polyphenol content and highest antioxidant activity [44,45]. According to Dorman and co-workers [46], eriocitrin and rosmarinic acid constitute the largest percentage (37.6% and 37.4%, respectively) of the peppermint polyphenol fraction. In our study, eriocitrin, luteolin-7-*O*-rutinoside, and rosmarinic acid had the largest percentages (54.7%, 20.3%, and 8.8%, respectively). Numerous reports on the multidimensional action, important for the proper functioning of the body, of both above-mentioned groups of compounds occurring in *Menthae piperitae folium*—flavonoids (especially glycosides of eriodictyol and luteolin) and phenolic acids (mainly rosmarinic acid)—are changing the current view on proper assessment of the validity of active substance in the raw material. While flavonoids are a well-known subgroup of polyphenols that have been used in medicine for years, phenolic acids are gradually being studied. In this research, quantitative analysis has shown the presence of 35 compounds (among them flavones, flavanones, phenolic acids—caffeetannins, lignin and jasmonic acid derivatives) in commercial peppermint tinctures, and 10 of them were determined quantitatively. Rosmarinic acid is a widely investigated phenolic acid that has gained great interest among pharmacologists [47]. Amoah and co-workers [16] published a comprehensive review in which they analyzed the entire scope of potential use of rosmarinic acid in various branches of medicine and highlighted its pharmacological and biological activities; among them, antioxidant, anti-inflammatory, anti-infective, and neuroprotective actions were described. In addition to caffeic and rosmarinic acids, 10 other caffeetannins were detected in the analyzed medicinal products by U(H)PLC-ESI-MS: salvianic acid A (danshensu), lithospermic acid A and its isomer, lithospermic acid B (salvianolic acids B) with three isomers, didehydrosalvianolic acid B, salvianolic acid C, and ethyl rosmarinate (an ester of rosmarinic acid with ethanol, the solvent used to prepare the tincture, probably an artefact). Generally oligomeric caffeic acid esters in peppermint tinctures were present at lower concentrations, below 0.45 mg/mL (Table A1), and the highest content was assessed for RA. Lithospermic acids A and B (and their isomers) were previously described in peppermint leaf infusions and other water extracts [38,39,48]. According to the authors’ data [38], lithospermic acid B makes up 1.0%–9.7% of polyphenol content. It was reported that this compound was an efficient free-radical scavenger that could be used to treat cardiovascular diseases and play a beneficial role in Alzheimer’s treatment [49,50]. The presence of didehydrosalvianolic acid B in peppermint leaf was reported previously by Krzyzanowska and co-workers [36]. On the basis of pseudo-molecular and fragmentation ions and literature data, one representative of lignans, medioresinol glucuronide, was identified in the analyzed samples [38].

To our knowledge, salvianolic acid C (previously found in *Mentha pulegium* L.), tuberonic acid sulphate, and tuberonic acid *O*-glucoside (present in *Mentha pulegium* L. and *Mentha longifolia* L.) have not been described in *Mentha* × *piperita* L. [35,40]. 

Currently peppermint leaf activity is attributed only to essential oil—a group of monoterpenes and their derivatives—as the main chemical parameter conditioning the multidirectional pharmacological action of the raw material and peppermint tincture. Polyphenols are found in the volatile fraction in negligible amounts. The content of essential oil in peppermint leaf is from 0.5%–4.5%, while the polyphenolic compounds constitute 19%–23% [28,51,52]. Olennikov and Tanakhaeva [53] conducted a quantitative determination of polyphenolic compounds using four spectrophotometric methods in *Mentha* × *piperita* L. originating in Siberia. Hesperidin and rosmarinic acid were used as standards. The authors concluded that the sum of polyphenols and monoterpenes contributes to the medicinal properties of peppermint [53]. The research suggests that the current pharmacopoeia requirement for *Menthae piperitae folium* should be corrected. In the pharmacopeial parameters required for admission of peppermint leaf to the market, a lower limit for the sum of polyphenols or the main biologically active components of the non-volatile fraction should be specified. According to our results as well as previous reports, the highest concentration compounds among peppermint polyphenols are eriocitrin, luteolin-7-*O*-rutinoside, and rosmarinic acid. Our study also showed that all polyphenolic compounds occur in analyzed commercial tinctures in extremely different concentrations. According to this, it is necessary to introduce to the pharmacopeial definition of *Menthae piperitae folium* (Ph. Eur.) and *Menthae piperitae tincture cum Menthae piperitae aetheroleo* (FP) corrections regarding the requirement for minimum content not only for rosmarinic acid but also for flavonoids or their leading representatives (e.g., eriocitrin and luteolin glycosides). From a pharmaceutical point of view, the presence of an unspecified, and thus unknown content of the active substance is an unacceptable oversight. Considering our research results, it can be assumed that one patient receives peppermint tincture no. 1 with the maximum contents of rosmarinic acid (0.44 mg/mL) and eriocitrin (1.63 mg/mL), and the other tincture no. 14 with the minimum contents of these compounds (0.01 and 0.09 mg/mL), respectively.

## 4. Materials and Methods 

### 4.1. Solvents and Chemicals

Reagents used in the analysis were of analytical grade. Acetonitrile (HPLC gradient grade), methanol, and 90%–100% formic acid were purchased from Sigma-Aldrich (St. Louis, MO, USA). Water was obtained in the process of distillation and deionization with Hydrolab Deionizer HLP20UV. U(H)PLC-ESI-MS grade water and formic acid were from Merck (Darmstadt, Germany); U(H)PLC-ESI-MS grade acetonitrile was from Honeywell (Morris Plains, NJ, USA). Analytical grade methanol for dilution was from POCh (Lublin, Poland). 

### 4.2. Reference Compounds and Standard Solutions

There were 20 standard solutions of polyphenols (flavonoids and phenolic acids) used in HPLC analysis. Commercial standards: eriodictyol (E), naringenin, hesperetin, apigenin, luteolin (L), luteolin-7-*O*-β-glucoside (Lg), apigenin-7-*O*-β-glucoside, rhoifolin (apigenin-7-*O*-neohesperidoside), isorhoifolin (Ir, apigenin-7-*O*-rutinoside), naringin (naringenin-7-*O*-neohesperidoside), narirutin (Nr, naringenin-7-*O*-rutinoside), hesperidin (Hr, hesperetin-7-*O*-rutinoside), diosmin (Dr, diosmetin-7-*O*-rutinoside), and rosmarinic acid (RA) were purchased from Extrasynthese (Genay, France); caffeic acid (CA) was purchased from Koch-Light Laboratories (Haverhill, UK); chlorogenic acid was purchased from Carl Roth (Karlsruhe, Germany). Eriocitrin (Er, eriodictyol-7-*O*-rutinoside) and luteolin-7-*O*-rutinoside (Lr) were isolated from peppermint leaves [54], and luteolin-7-*O*-β-glucuronide (Lgr) and lithospermic acid A (LAA) were isolated from *Serpylli herba* [43].

Stock standard solutions (1 mg/mL) were prepared by dissolving a weighted amount (precisely about 1.0–1.7 mg) of standard in the appropriate methanol volume (1.0–1.7 mL) and filtered through a 0.45 µm membrane filter (Millipore, Burlington, MA, USA). Working standard solutions, with concentrations of 0.02, 0.05, 0.1, 0.15, 0.2, and 0.3 mg/mL, were obtained by diluting stock standard solutions with 50% aq. methanol (*v/v*). Working standard solutions were used for standard curve preparation. 

In addition to the above-mentioned compounds, the following infusions (1:20, *m/v*) from herbal reference materials *Melissa officinalis* L. (leaves) and *Salvia miltiorrhiza* Bunge (roots) were used in U(H)PLC-ESI-MS analysis.

### 4.3. Material and Sample Preparation

Twenty-three different commercial peppermint tinctures (*Menthae piperitae tinctura*) with different batch numbers were purchased from pharmacies from 2016–2019. Those traditional herbal medicines were manufactured in Poland by Hasco-Lek Wroclaw, Herbapol Lublin SA, Herbapol Kraków, and Amara Pharmaceutical Industries Kraków. Peppermint tinctures were diluted with 50% methanol (*v/v*) in a 1:5 ratio for the purpose of HPLC-DAD and in a 1:20 ratio for U(H)PLC-ESI-MS analysis. Diluted solutions were filtered through a 0.45 µm membrane filter (Millipore, Burlington, MA, USA). Qualitative analysis was confirmed by the U(H)PLC-ESI-MS method. Quantitative and qualitative analysis were performed with the HPLC-DAD method. 

### 4.4. U(H)PLC-ESI-MS Conditions 

Confirmation of analyzed polyphenols’ identity was achieved through mass spectrometry coupled with liquid chromatography. The Thermo Scientific U(H)PLC Ultimate 3000 apparatus (Thermo Fisher Scientific, Waltham, MA, USA) comprised a set of LPG-3400RS quaternary pumps with a vacuum degasser, WPS-3000TRS autosampler, TCC-3000SD column oven, and DAD-3000 photodiode detector. The HRMS (High Resolution Mass Spectrometry) detector was an ESI-qTOF Compact (Bruker Daltonics, Bremen, Germany). The separation was performed on a Kinetex RP-18 column (150 × 2.1 mm i.d., octadecyl ∅ 2.6 µm; Phenomenex, Torrance, CA, USA), equipped with a dedicated precolumn. The U(H)PLC-ESI-MS instrument was operated in both negative and positive modes in separate runs. The HRMS detector was calibrated in the dead time of every run with the Tunemix mixture (Bruker Daltonics) with *m/z* standard deviation below 0.5 ppm. The resulting mass spectra analysis was carried out using Data Analysis 4.2 software (Bruker Daltonics). The ions were collected in the scan range 50–2200 *m/z* with low mass set at 150 *m/z*. The important instrument parameters were as follows: for source—nebulizer pressure 1.5 bar, dry gas N_2_ with flow 7.0 L/min, temperature 200 °C, capillary voltage 2.2 kV; for quadrupole—ion energy 5 eV; for collision cell—collision gas N_2_, collision energy in basic MS mode 10 eV, in auto MS/MS mode performed for ions in the range 400–1900 *m/z*. The gradient elution system consisted of 0.1% formic acid in water (mobile phase A) and 0.1% formic acid in acetonitrile (mobile phase B). At the flow rate of 0.3 mL/min, the following elution program was used: 0–1 min (2% B in A), 1–31 min (2→100% B in A), 31–36 min (100% B in A). The column was equilibrated for 5 min before the next analysis. Blanks were run after each sample to avoid cross-contamination. Other parameters were: column oven temperature 30 °C, injection volume 5 µL. 

### 4.5. HPLC-DAD Conditions 

HPLC-DAD analysis was performed on a Dionex ThermoScientific Ultimate 3000 high performance liquid chromatograph equipped with a LPG-3400D pump, WPS-3000TSL autosampler, column thermostat, and diode array detector UV DAD-3000. Hardware was controlled with Chromeleon Software. Analysis was conducted with a Thermo Hypersil Column (250 mm × 4.6 i.d., octadecyl Beta Basic-18 ∅ 5µm, Loughborough, UK). UV detection was performed during the analysis with three wavelengths: 280 nm (flavanone derivatives), 320 nm (caffeic acid derivatives), and 360 nm (flavone derivatives). Polyphenolic compounds were analyzed with the following eluents: C, water–formic acid (95.0:5.0 *v/v*) and D, acetonitrile–formic acid (95.0:5.5 *v/v*). The addition of formic acid prevented polyphenolic acid dissociation. HPLC gradient: 0–25 min (7→30% D in C), 25–30 min (30→70% D in C), 30–35 min (70→7% D in C). The analysis was conducted at 20 °C with sample injection of 20 µL, and the eluent flowrate was maintained at 0.9 mL/min. This method was developed and validated by Fecka and Turek [43].

### 4.6. Content Measurement

The content of individual polyphenols and the sums of particular phytochemical groups (flavonoids and phenolic acids) were determined using the optimized HPLC-DAD method [43]. Calibration equations for quantified flavonoids and phenolic acids were assessed at six concentration levels, and duplicate injections were performed for each concentration (*n* = 2 × 6). The linearity of standard curves was confirmed by plotting the peak areas (*y*, mAU/s) and the corresponding concentration (*x*, mg/mL). Limits of detection (LOD) and limits of quantitation (LOQ) were calculated from calibration equations based on the signal-to-noise ratio (S/N ≥ 3:1 and S/N ≥ 10:1) and expressed as the concentration of the examined compound (mg/mL). Table 3 presents chromatographic characteristics of standard compounds (calibration equation, r^2^, range, LOD, LOQ, working wavelength).

## 5. Conclusions

For the first time it is noted that flavonoids and phenolic acids (caffeetannins), the main components of the non-volatile peppermint fraction which play an important role in the pharmacological action of this medicinal plant, have not yet found a place next to essential oil in the pharmacopeial definition of peppermint leaf (*Menthae piperitae folium*, Ph. Eur.) and peppermint tincture (*Menthae piperitae tinctura cum Menthae piperitae aetheroleo*, FP). Based on the results of this research as well as data from the literature, the chemical composition of peppermint tincture should be carefully analyzed and standardized. In the light of these results, it seems that the pharmacopeial assessment for *Menthae piperitae folium* and *Menthae piperitae tinctura cum Menthae piperitae aetheroleo* requires correction and supplementation.

## Figures and Tables

**Table 1:** LC-HRMS, UV-Vis, and MS/MS properties of compounds identified in peppermint tinctures.

Peak No.	t_R_ (min)	[M-H]^−^ (*m/z*, meas.)	Err. (ppm)	[M-H]^−^ (formula)	MS/MS Fragments (*m/z*)	UV_max_ (nm)	Identification Proposal	Reference
**Phenolic acids—caffeetannins**								
**1**	**1.70**	197.0454	0.9	C_9_H_9_O_5_	181, 137	-	Salvianic acid A (danshensu)	[30]
**3**	**9.25**	179.0353	−1.7	C_9_H_7_O_4_	-	290, 325	Caffeic acid	s
**7**	**11.33**	537.1048	−1.8	C_27_H_21_O_12_	295	285, 315, 343	Salvianolic acid J/W or other isomer	[31,32,33]
**15**	**12.26**	717.1442	2.6	C_36_H_29_O_16_	475, 431, 365, 339, 321, 295	280, 320	Salvianolic acid E/L or other isomer	[30,32]
**19**	**12.58**	717.1440	3.0	C_36_H_29_O_16_	339, 321, 295	285, 335	Salvianolic acid E/L or other isomer	[30,32]
**21**	**12.90**	359.0783	−3.1	C_18_H_15_O_8_	191, 161	290, 328	Rosmarinic acid	s
**22**	**13.01**	537.1040	−0.3	C_27_H_21_O_12_	493, 295, 185	254, 290, 310	Lithospermic acid A	s
**24**	**13.35**	717.1455	0.9	C_36_H_29_O_16_	519, 339, 321, 295	-	Lithospermic acid B (salvianolic acid B)	rm [34]
**25**	**13.53**	717.1392	9.7	C_36_H_29_O_16_	-	-	Lithospermic acid B isomer	
**27**	**14.09**	491.0988	-0.8	C_26_H_19_O_10_	311, 293	320	Salvianolic acid C	[30,35]
**30**	**14.71**	715.1293	1.6	C_36_H_27_O_16_	337, 319, 293	280, 340	Didehydrosalvianolic acid B	[36]
**34**	**16.15**	387.1092	−1.8	C_20_H_19_O_8_	359	-	Ethyl rosmarinate	[37]
**Flavones**								
**5**	**10.44**	637.1053	−1.0	C_27_H_25_O_18_	285	265, 345	Luteolin-*O*-diglucuronide	[38]
**9**	**11.42**	593.1513	−0.1	C_27_H_29_O_15_	285	265, 350	Luteolin-7-*O*-rutinoside	s
**10**	**11.71**	447.0930	0.6	C_21_H_19_O_11_	285	270, 345	Luteolin-7-*O*-β-glucoside	s
**12**	**11.77**	461.0716	2.0	C_21_H_17_O_12_	285	270, 348	Luteolin-7-*O*-β-glucuronide	s
**14**	**12.18**	577.1558	0.8	C_27_H_29_O_14_	269	268, 335	Apigenin-7-*O*-rutinoside (isorhoifolin)	
**17**	**12.46**	607.1668	0.0	C_28_H_30_O_15_	299, 284	270, 340	Diosmetin-7-*O*-rutinosidee (diosmin)	s
**18**	**12.57**	431.0979	1.2	C_21_H_19_O_10_	-	267, 335	Apigenin-7-*O*-glucoside	s
**20**	**12.67**	445.0762	3.2	C_21_H_17_O_11_	-	267, 335	Apigenin-7-*O*-glucuronide	
**23**	**13.08**	461.0723	0.5	C_21_H_17_O_12_	-	280, 320	Tetrahydroxyflavone-*O*-glucuronide (e.g., scutellarin)	
**28**	**14.12**	489.1037	0.2	C_23_H_21_O_12_	-	-	Luteolin-7-*O*-glucuronide ethyl ester	
**31**	**14.80**	285.0403	0.5	C_15_H_9_O_6_	-	-	Luteolin	s
**33**	**16.10**	269.0456	−0.2	C_15_H_9_O_5_	-	-	Apigenin	s
**Flavanones**								
**6**	**11.13**	595.1672	−0.6	C_27_H_31_O_15_	287	285	Eriodictyol-7-*O*-rutinoside (eriocitrin)	s
**8**	**11.42**	449.1088	0.2	C_21_H_21_O_11_	287	285	Eriodictyol-7-*O*-glucoside	[39]
**11**	**11.73**	463.0862	4.3	C_21_H_19_O_12_	287		Eriodictyol-7-*O*-glucuronide	[38]
**13**	**11.97**	579.1709	1.7	C_27_H_31_O_14_	271	280	Naringenin-7-*O*-rutinoside (narirutin)	s
**16**	**12.41**	609.1819	1.0	C_28_H_33_O_15_	301, 286	285	Hesperetin-7-*O*-rutinoside (hesperidin)	s
**29**	**14.41**	287.0563	−0.5	C_15_H_11_O_6_	-	-	Eriodictyol	s
**32**	**15.91**	271.0618	−2.1	C_15_H_11_O_5_	-	-	Naringenin	s
**35**	**16.35**	301.0725	−2.3	C_16_H_13_O_6_	-	-	Hesperetin	s
**Jasmonic acid derivatives**								
**2**	**9.14**	305.0700	0.1	C_12_H_17_O_7_S	-	285, 320	Tuberonic acid sulphate	[40]
**4**	**9.39**	387.1661	−0.4	C_18_H_27_O_9_	-	280, 315	Tuberonic acid *O*-glucoside	[40]
**Lignans**								
**26**	**13.99**	563.2124	1.8	C_28_H_35_O_12_	387	280, 320	Medioresinol *O*-glucuronide	[40]

s—identification conducted using authentic standards; rm, reference material—*Salvia miltiorrhiza*; In other cases, appropriate literature reference used in identification is indicated. LC-HRMS, liquid chromatography-high resolution mass spectrometry.

**Table 2:** Analysis of fragmentation behaviors of trimeric and tetrameric caffeetannins.

Pseudo-Molecular Ion:	Fragment Ion	Interpretation		Reference
**717:**	519 [M-198 − H]^−^	[M-198 − H]^−^ [M-180-18 − H]^−^		[34]
	475 [M-242 − H]^−^	[M-198-44 − H]^−^ [M-180-44-18 − H]^−^		[34,35]
	431 [M-286 − H]^−^	[M-198-44-44 − H]^−^ [M-180-44-44-18 − H]^−^		
	339 [M-378 − H]^−^	[M-198-180 − H]^−^ [M-180-180-18 − H]^−^		[34]
	321 [M-396 − H]^−^	[M-198-198 − H]^−^ [M-198-180-18 − H]^−^ [M-180-180-18-18 − H]^−^		
	295 [M-422 − H]^−^	[M-198-180-44 − H]^−^ [M-180-180-44-18 − H]^−^		[34]
**715:**	337 [M-378 − H]^−^	[M-198-180 − H]^−^ [M-180-180-18 − H]^−^		[36]
	319 [M-396 − H]^−^	[M-198-198 − H]^−^ [M-198-180-18 − H]^−^ [M-180-180-18-18 − H]^−^		
	293 [M-422 − H]^−^	[M-198-180-44 − H]^−^ [M-180-180-44-18 − H]^−^		
**537:**	493 [M-44 − H]^−^	[M-44 − H]^−^		[34]
	295 [M-242 − H]^−^	[M-198-44 − H]^−^ [M-180-44-18 − H]^−^		
**491:**	311 [M-180 − H]^−^	[M-180 − H]^−^		[34]
	293 [M-198 − H]^−^	[M-198 − H]^−^ [M-180-18 − H]^−^		
Neutral loss of:				
198 Da →	salvianic acid A = danshensu (C_9_H_10_O_5_)		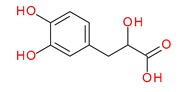	
180 Da →	dehydroxysalvianic acid A residue = dehydroxydanshensu residue (C_9_H_8_O_4_) ↓ caffeic acid (C_9_H_8_O_4_)		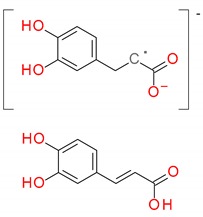	
44 Da →	CO_2_			
18 Da →	H_2_O			

**Table 3:** Validation parameters of standard compounds for high performance liquid chromatography coupled with a diode-array detector (HPLC-DAD) analysis.

Standard	Calibration Equation	*r* ^2^	Linear Range (µg/mL)	LOD (µg/mL)	LOQ (µg/mL)	Working Wavelength
Flavones						
Luteolin	y = 1224.6x − 0.1205	0.9990	10–300	0.245	0.817	360
Luteolin-7-*O*-β-glucuronide	y = 615.69x − 0.9761	0.9996	10–250	0.487	1.624	360
Luteolin-7-*O*-β-rutinoside	y = 457.74x − 0.9438	0.9998	10–250	0.655	2.185	360
Isorhoifolin	y = 875.93x − 0.2331	0.9996	10–250	0.343	1.142	360
Flavanones						
Narirutin	y = 294.76x + 0.675	0.9998	10–250	1.018	3.393	280
Eriodictyol	y = 330.28x + 0.2099	0.9998	10–250	0.908	3.028	280
Eriocitrin	y = 239.18x + 0.8709	0.9992	10–300	1.254	4.189	280
Hesperidin	y = 480.26x + 0.2943	0.9995	10–300	0.625	2.082	280
Phenolic acids—Caffeetannins						
Caffeic acid	y = 1824.74x − 4.64	0.9993	10–300	0.164	0.548	320
Rosmarinic acid	y = 581.91x + 1.99	0.9991	10–300	0.515	1.718	320

LOD, limit of detection; LOQ, limit of quantification.

## Appendix A

**Table A1:** Content of determined polyphenolic compounds in peppermint tinctures (mg/mL).

No.	CA.	RA	SPA	Er	Lr ^1^	Lgr	Nr	Ir	Hr ^2^	E	L	SF	SPP
1	0.01 ^a^	**0.44** ^c^	**0.45** ^c^	1.63 ^c^	0.27 ^b^	0.03 ^d^	0.00	0.00	0.09 ^b^	0.01 ^c^	0.00	2.03 ^c^	2.48 ^c^
2	0.01 ^a^	0.22 ^b^	0.23 ^b^	1.70 ^a^	0.71 ^a^	0.04 ^b^	0.08 ^b^	0.00	0.09 ^c^	0.01 ^c^	0.00	2.61 ^a^	2.84 ^a^
3	0.01 ^a^	0.01 ^c^	0.02 ^b^	0.18 ^a^	*0.06* ^d^	0.00	0.00	0.00	0.01 ^a^	0.00	0.00	0.26 ^b^	0.28 ^b^
4	0.02 ^a^	0.21 ^b^	0.22 ^b^	1.76 ^b^	0.66 ^b^	0.03 ^d^	0.17 ^c^	0.00	0.05 ^c^	0.01 ^b^	0.01 ^c^	2.68 ^b^	2.91 ^b^
5	0.02 ^b^	0.38 ^b^	0.39 ^b^	1.46 ^b^	0.36 ^b^	**0.05** ^d^	0.07 ^c^	0.00	**0.10** ^d^	0.01	0.01 ^a^	2.06 ^a^	2.45 ^a^
6	0.02 ^b^	0.24 ^a^	0.26 ^a^	1.04 ^a^	0.32 ^b^	0.02 ^d^	0.09 ^b^	0.00	0.07 ^b^	*0.00*	0.01 ^d^	1.54 ^b^	1.80 ^a^
7	0.02 ^b^	0.22 ^a^	0.23 ^a^	**1.77** ^a^	0.67 ^a^	0.03 ^b^	0.19 ^d^	0.00	0.06 ^a^	0.00	0.01 ^c^	2.72 ^a^	2.95 ^a^
8	0.02 ^b^	0.22 ^b^	0.24 ^b^	1.76 ^b^	**0.74** ^b^	0.04 ^c^	**0.23** ^d^	0.00	0.08 ^b^	0.00	0.01 ^a^	**2.85** ^b^	**3.09** ^b^
9	0.02 ^a^	0.13 ^a^	0.15 ^a^	0.99 ^b^	0.21 ^a^	0.02 ^d^	0.03 ^b^	0.01 ^c^	0.06 ^c^	0.01 ^d^	0.00	1.33 ^b^	1.48 ^c^
10	0.01 ^a^	0.13 ^b^	0.14 ^b^	1.09 ^a^	0.35 ^a^	0.04 ^c^	0.07 ^a^	**0.03** ^c^	0.04 ^b^	0.01 ^a^	0.00	1.62 ^a^	1.76 ^a^
11	0.02 ^a^	0.15 ^a^	0.16 ^a^	0.72 ^a^	0.24 ^a^	0.04 ^a^	0.02 ^b^	0.01 ^a^	0.05 ^b^	0.06 ^a^	0.00	1.14 ^a^	1.31 ^a^
12	0.01 ^a^	0.11 ^a^	0.12 ^d^	0.82 ^d^	0.25 ^c^	0.02 ^a^	0.03 ^a^	0.01 ^a^	0.04 ^a^	**0.10** ^d^	0.02 ^a^	1.29 ^a^	1.41 ^c^
13	0.00	0.02 ^a^	0.02 ^a^	0.19 ^d^	0.08 ^a^	0.01 ^a^	0.01 ^a^	0.00	0.01 ^a^	0.03 ^a^	0.01 ^a^	0.34 ^c^	0.36 ^c^
14	0.00	*0.01* ^a^	*0.01* ^a^	*0.09* ^a^	0.07 ^a^	0.01 ^a^	0.00	0.00	0.01 ^a^	0.03 ^a^	0.02 ^a^	*0.23* ^a^	*0.24* ^a^
15	0.01 ^a^	0.08 ^a^	0.09 ^d^	0.48 ^c^	0.17 ^c^	0.02 ^a^	*0.00*	0.00	0.04 ^a^	0.03 ^a^	0.03 ^a^	0.77 ^b^	0.86 ^b^
16	0.00	0.02 ^a^	0.02 ^a^	0.16 ^a^	0.10 ^c^	0.01 ^a^	0.01 ^a^	0.00	0.02 ^a^	0.04 ^a^	0.04 ^a^	0.38 ^c^	0.40 ^c^
17	0.00	0.04 ^a^	0.04 ^a^	0.34 ^c^	0.08 ^c^	0.01 ^a^	0.01 ^a^	0.00	0.02 ^a^	0.04 ^a^	*0.00*	0.50 ^c^	0.54 ^d^
18	0.00	0.03 ^a^	0.03 ^a^	0.18 ^a^	0.12 ^a^	0.01 ^a^	0.01 ^a^	0.00	0.02 ^a^	0.05 ^a^	0.04 ^a^	0.43 ^a^	0.46 ^a^
19	0.00	0.01 ^a^	0.01 ^a^	0.10 ^c^	0.07 ^c^	0.01 ^a^	0.01 ^a^	0.00	*0.01* ^a^	0.03 ^a^	0.00	0.23 ^c^	0.24 ^c^
20	0.01 ^a^	0.25 ^c^	0.26 ^c^	0.64 ^c^	0.22 ^c^	0.03 ^a^	0.02 ^a^	0.01 ^a^	0.04 ^a^	0.03 ^a^	0.01 ^a^	1.00 ^c^	1.26 ^b^
21	0.01 ^a^	0.20 ^d^	0.21 ^d^	0.94 ^c^	0.24 ^c^	0.03 ^a^	0.02 ^a^	0.01 ^a^	0.08 ^c^	0.04 ^a^	0.01 ^a^	1.37 ^d^	1.58 ^d^
22	0.00	0.06 ^d^	0.06 ^d^	0.44 ^c^	0.15 ^d^	0.02 ^a^	0.01 ^a^	0.00	0.03 ^a^	0.05 ^c^	0.01 ^a^	0.71 ^d^	0.77 ^c^
23	0.00	0.02 ^a^	0.02 ^d^	0.13 ^a^	0.09 ^c^	0.01 ^a^	0.01 ^a^	0.00	0.01 ^a^	0.06 ^a^	**0.04** ^a^	0.35 ^c^	0.37 ^b^
MEAN	***0.01***	***0.14***	***0.15***	***0.81***	***0.27***	***0.02***	***0.05***	***0.00***	***0.04***	***0.03***	***0.01***	***1.24***	***1.39***
SD	*0.00*	*0.12*	*0.12*	*0.60*	*0.23*	*0.01*	*0.06*	*0.01*	*0.03*	*0.02*	*0.01*	*0.87*	*0.97*
MED	*0.01*	*0.13*	*0.14*	*0.72*	*0.22*	*0.02*	*0.02*	*0.00*	*0.04*	*0.03*	*0.01*	*1.14*	*1.31*
MAX	*0.02*	*0.44*	*0.45*	*1.77*	*0.74*	*0.05*	*0.23*	*0.03*	*0.10*	*0.10*	*0.04*	*2.85*	*3.09*
MIN	*0.00*	*0.01*	*0.01*	*0.09*	*0.06*	*0.00*	*0.00*	*0.00*	*0.01*	*0.01*	*0.00*	*0.23*	*0.24*

^1^ Lgr(+Lg) calculated as Lgr, ^2^ Hr(+Dr) calculated as Hr, ^a^ less than 1% CV ^b^ less than 3% CV, ^c^ less than 5% CV, ^d^ less than 7% CV; n = 3.
